# Scalable Sacrificial Templating to Increase Porosity and Platinum Utilisation in Graphene-Based Polymer Electrolyte Fuel Cell Electrodes

**DOI:** 10.3390/nano11102530

**Published:** 2021-09-28

**Authors:** Theo A. M. Suter, Adam J. Clancy, Noelia Rubio Carrero, Marie Heitzmann, Laure Guetaz, Paul R. Shearing, Cecilia Mattevi, Gérard Gebel, Christopher A. Howard, Milo S. P. Shaffer, Paul F. McMillan, Dan J. L. Brett

**Affiliations:** 1 Electrochemical Innovation Lab, Department of Chemical Engineering, University College London, London WC1H 0AJ, UK; p.shearing@ucl.ac.uk; 2 Department of Chemistry, University College London, London WC1H 0AJ, UK; a.clancy@ucl.ac.uk (A.J.C.); p.f.mcmillan@ucl.ac.uk (P.F.M.); 3 Department of Physics and Astronomy, University College London, London WC1H 0AJ, UK; c.howard@ucl.ac.uk; 4 Department of Chemistry, Imperial College London, London SW7 2AZ, UK; n.rubio-carrero@imperial.ac.uk (N.R.C.); m.shaffer@imperial.ac.uk (M.S.P.S.); 5 Department of Materials, Imperial College London, London SW7 2AZ, UK; c.mattevi@imperial.ac.uk; 6 Université Grenoble Alpes, CEA, Liten, DEHT, 38000 Grenoble, France; marie.heitzmann@cea.fr; 7 Université Grenoble Alpes, CEA, Liten, DTNM, 38054 Grenoble, France; laure.guetaz@cea.fr (L.G.); gerard.gebel@cea.fr (G.G.)

**Keywords:** fuel cells, graphene, porosity, electrode structure, spacers

## Abstract

Polymer electrolyte fuel cells hold great promise for a range of applications but require advances in durability for widespread commercial uptake. Corrosion of the carbon support is one of the main degradation pathways; hence, corrosion-resilient graphene has been widely suggested as an alternative to traditional carbon black. However, the performance of bulk graphene-based electrodes is typically lower than that of commercial carbon black due to their stacking effects. This article reports a simple, scalable and non-destructive method through which the pore structure and platinum utilisation of graphene-based membrane electrode assemblies can be significantly improved. Urea is incorporated into the catalyst ink before deposition, and is then simply removed from the catalyst layer after spraying by submerging the electrode in water. This additive hinders graphene restacking and increases porosity, resulting in a significant increase in Pt utilisation and current density. This technique does not require harsh template etching and it represents a pathway to significantly improve graphene-based electrodes by introducing hierarchical porosity using scalable liquid processes.

## 1. Introduction

Polymer electrolyte fuel cells (PEFCs) are an electrochemical energy conversion technology that is key to helping decarbonise the transport, portable, and stationary power sectors [1,2,3]. However, there are cost and technical challenges that must be overcome before this technology sees significantly wider commercial use. Catalysts are required to achieve high current densities for the hydrogen oxidation/oxygen reduction reactions (HOR/ORR) at the anode/cathode sites, respectively. The sluggish ORR reaction at the cathode is currently one of the main barriers impeding device performance. Platinum (Pt) and Pt-alloy nanoparticles are by far the most effective ORR catalysts; however, their low earth-abundance and expensive processing limit the commercial uptake of this technology [4,5,6]. Much research is ongoing into the development of non-Pt ORR catalysts [7,8,9,10] and the optimization of Pt-based catalysts. Conversely, the role of the catalyst layer (CL) structure in defining catalyst accessibility and cell power density is often overlooked [9]. The CL structure can be designed to optimise catalyst accessibility and reduce mass transport resistance by tuning the porosity, adopting novel substrate architectures, or by the use of unsupported CLs [11,12,13,14]. Such performance enhancements resulting from CL design are expected to lead to improved MEAs for both optimised Pt-based catalysts and emerging alternatives.

Fuel cell CLs are typically 2–20 μm thick and consist of electrically-conducting carbon supports decorated with Pt-based nanoparticle catalysts. They utilise proton-conducting polymers (referred to as ionomer) as both binder and electrolyte, which in turn are in contact with the proton-conducting polymer membrane [6,15]. To maximise current density and effective Pt utilisation, the ionomer network and the electrically conductive carbon pathways must maintain high conductivity while allowing efficient mass transport by maintaining high porosity. The power density of a fuel cell is dependent on achieving a balance between proton and electronic conduction, reactant (O_2_ and H_2_) transport and water management [16,17]. Thus, the engineering of the CL structure plays a critical role in controlling both power output and durability under operational conditions [18,19,20,21,22]. The chemical nature of the support material defines the intrinsic electronic conductivity and corrosion resistance. The morphology of the CL (its porosity, pore size distribution, texture and connectivity) is also impacted by the size and shape of the conductive support. The most widely adopted catalyst support materials, which are both used in academic research and implemented commercially, are carbon blacks, which provide high electrical conductivity, a low cost, and a surface area ranging between 200–1000 m^2^ g^−1^ [23,24]. High surface area carbons (HSAC) typically possess a high microporosity and are less graphitic than low surface area carbons (LSAC). The reduced graphitisation and the presence of surface oxygen functional groups in HSAC make them highly susceptible to oxidative damage, limiting fuel cell durability [25,26]. Carbon corrosion of the support is one of the main degradation mechanisms for an active fuel cell, and occurs faster under fuel starvation or high humidity operations that are common in real-world applications [25,27].

**Figure 1:**
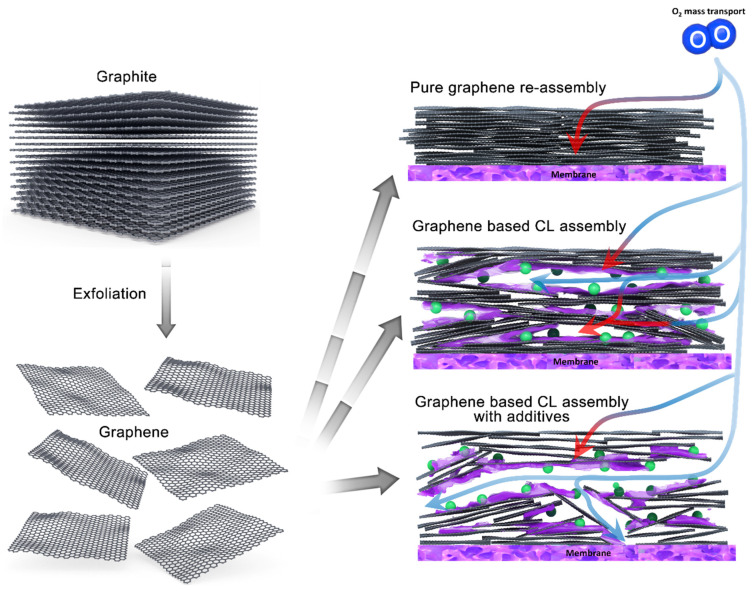
Schematic showing the process of fabricating graphene-based CLs, and its effects on the oxygen transport pathways from the flow field across the CL. Accessible gas pathways are indicated by blue arrows, while inaccessible gas pathways are indicated by red arrows. Typical graphene-based CL form low porosity electrodes inhibit the flow of oxygen throughout the CL, reducing Pt utilisation and increasing mass transport resistances. The pore structure of the CL can be modified via the use of spacers or additives to widen the pore network, increasing Pt utilisation.

Graphene has been widely proposed as an attractive catalyst support material, mainly due to its sp^2^-bonded framework, which is significantly more resistant to oxidation than carbon black [16,17,28,29]. Graphene’s light weight, high intrinsic surface area, and excellent electrical conductivity are also desirable features. While the theoretical surface area of true, single-layer graphene is very high (2630 m^2^ g^−1^), in practical bulk systems, the ‘graphene’ used is rarely fully exfoliated. Graphene-based CLs are typically formed from dispersions of few-layer graphenes (FLGs), which have surface areas of only a few hundred metres squared per gram. Furthermore, 2D sheets are prone to restacking during deposition, which dramatically reduces their accessible surface area and Pt utilisation, due to the catalyst particles becoming trapped between the layers [30,31,32]. The stacking of graphene layers results in thick, low-porosity electrodes that significantly limit power density by increasing the tortuosity of the gas diffusion bath, or blocking it altogether (Figure 1). The stacking limitation is exacerbated by a lack of interior micro/meso pores, which are intrinsic to commercial carbon blacks [30,33,34,35]. While micropores in carbon blacks account for a significant portion of CL porosity and surface area, mesopores are often reported as optimum sites for the deposition of electrochemically-active Pt nanoparticles [23,36]. Micropores are defined as pores smaller than 2 nm [24] and mesopores as pores between 2 and 50 nm, which can be due to larger channels within a particle or to the void space between particles [23,24,36]. Macropores (pores larger than 50 nm) have been referred to as O_2_ diffusion highways as they are generally regarded as being key to low-tortuousity gas diffusion, particularly in thicker CLs [37,38]. The optimisation of the pore network of the carbon support has been reported as having improved the electrochemical surface area (ECSA) and the mass activity of the catalyst, due to the protection it offers from direct ionomer poisoning [23,39,40,41,42]. Since graphene has no inherent pore structure, the CL morphology is defined by the void spaces between the graphene sheets formed during electrode manufacturing. Advanced fabrication techniques are required to mitigate restacking and provide morphologically-optimised, graphene-based electrodes with competitive performance [31,43]. One of the possible advantages of an FLG-based CL is high mesoporosity without the need for microporosity.

While attempts have been made to manufacture porous graphene electrodes by novel techniques, such as freeze-drying, they typically use the less conductive and more defective variant of graphene, (reduced) graphene oxide, or they rely on a non-scalable manufacturing route [30,31,44,45]. As such, the performance and commercial relevance of graphene-based PEFC electrodes is limited by the CL morphology achievable with more graphitic feedstocks. Furthermore, it is non-trivial to establish the relative durability of graphene against carbon corrosion compared to carbon black if there are significant differences in Pt utilisation and current density. To maximise the performance of graphene-based electrodes in both the kinetic and mass transport regimes of a polarisation curve, a balanced pore structure needs to be engineered during the manufacturing stage. Importantly, the techniques used must be readily scalable for the technology to achieve widespread commercial usage.

The most widely reported technique for mitigating the propensity of graphene inks to re-stack and form low-porosity CLs when deposited is to introduce a second electrically-conducting carbon support material to act as a spacer [30,44,46,47]. Typically, the spacers are carbon-based materials with different morphologies to graphene, such as carbon black or carbon nanotubes. The introduction of these spacers forces the separation of the sheets of graphene in the CL, which helps to increase porosity and create a balanced pore distribution [44,48,49,50]. However, by introducing an additional carbon material with different surface chemistries, the reported benefits of carbon corrosion stability or scalability of graphene can be lost. Although CNTs should possess the same corrosion stability as graphene, their cost and difficulty in forming stable solutions make them non-ideal for spray deposition. It is, therefore, preferable to separate the graphene sheets and introduce porosity via a method that retains an entirely graphene-supported CL. Alternative manufacturing techniques, including colloid templating, have been suggested to improve performance by using a similar introduction of porosity and choice of pore size distribution (PSD), allowing for greater Pt utilization [51,52]. However, these approaches typically require the use of a destructive processing step, such as refluxing in NaOH or ionomer impregnation after CL assembly, which can result in a heterogenous proton conducting network. Alternatively, water-soluble sacrificial additives have been reported as offering improved performance when added to the ink before fabrication and removed after electrode assembly [11,12]. These studies used an unsupported Pt_3_Ni aerogel as a CL, in which K_2_CO_3_ additives were found to induce changes in the CL morphology, modifying the porosity and improving mass transport.

In this study, a simple, industrially-scalable and non-damaging method was developed that improved the pore structure and Pt utilisation. This method uses urea as a sacrificial additive to the standard aqueous-based CL ink, which, when deposited, forms water-soluble organic crystals physically separating the FLG sheets during deposition. Urea was chosen as it is low-cost, environmentally benign, and widely available; and unlike salt additives, it was found to not destabilise graphene ink suspension. The urea can then be removed by simple soaking in water, leaving voids between the FLG particles. This process maintains the structure of the graphitic component while introducing meso- and macroscale porosity to the CL, dramatically increasing current density. Due to the mildness of the treatment, the benefits are obtained without damaging the graphene sheets or disrupting the continuous ionomer network.

## 2. Materials and Methods

### 2.1. Materials

The graphene nanoplatelets (xGnP-M5, 5 μm particle size, surface area 120–150 m^2^ g^−1^), sodium metal (ingot, 99.95%), naphthalene (99%), dimethylacetamide (DMAc, anhydrous, 99.8%), 4-bromothioanisole (97%), chloroplatinic acid hydrate (≥99.9%, H_2_PtCl_6_.xH_2_O, assuming x = 2), sodium borohydride (≥98.0%), and urea (≥99.5%, powder, Merck) were supplied by Sigma Aldrich (Gillingham, UK). The acetone (99.8%) and propan-2-ol (99%) were supplied by VWR Ltd. (Luttleworth, UK). The Nafion^®^ Dispersion (Type DE-1021, Lot SGWO8-02SC) was obtained from DuPont Fluoroproducts (Richmond, VA, USA). The carbon black (Vulcan XC72R) and Freudenberg H23C7 was supplied by Fuel Cell Stores (College Station, TX, USA). The argon (99.95%) was supplied by BOC gases (Munich, Germany).

The water was deionised using an Elga PureLab Chorus on site. The DMAc was dried over 4 Å molecular sieves (ca. 15 vol.%, activated at 200 °C, 10 mbar, 12 h) for 48 h before use. The graphene nanoplatelets were dried under vacuum (200 °C, 10^−7^ mbar, 16 h) before use. All the other chemicals were used as supplied.

### 2.2. Synthesis

FLG Functionalisation. The procedure used was adapted from Clancy et al. [53]. In an argon glovebox, sodium (383 mg, 16.7 mmol) and naphthalene (2.13 g) were stirred in DMAc (2 dm^3^) with a glass stirrer bar until all the sodium dissolved, leaving a deep green solution (ca. 4 h) of sodium naphthalide. Graphene nanoplatelets (2 g, 167 mmol) were added and stirred (12 h) to form a black solution. 4-Bromoanisole (1.13 g, 5.56 mmol) was added to the solution (12 h) before removing the mixture from the glovebox, filtering it over filter paper and washing it with water and acetone to produce a black powder of functionalised few-layered graphite (f-FLG, ca. 2.1 g).

Platinum Nanoparticle Deposition. The chloroplatinic acid hydrate (1.08 g) and f-FLG (500 mg) were mixed with water (500 mL) and briefly bath-sonicated (15 min, 50 W) to produce a metastable suspension. The mixture was vigorously stirred and sodium borohydride solution (120 mL, 0.1 M aqueous) was rapidly added and left to stir (16 h). The mixture was then filtered to produce Pt@FLG.

Catalyst Ink Formulation. The graphene-based catalyst inks were prepared by mixing Pt@FLG (60 mg, 40wt% Pt), Nafion solution (260 µL, 11 wt% Nafion, DuPont Fluoroproducts (Richmond, VA, USA)., water (30 mL) and propan-2-ol (30 mL) in a 100 mL Mason Jar. The commercial Pt/C catalyst ink consisting of Pt/C (60 mg, 40wt% Pt, Vulcan XC72R, Alfa Aesar, (Ward Hill, Mass., USA), Nafion solution 11 wt% were supplied by DuPont Fluoroproducts (Richmond, VA, USA)., water (30 mL) and propan-2-ol (30 mL) were likewise prepared in a 100 mL Mason Jar. The ratio between the ionomer and the carbon support was 0.8:1. In the sacrificial templated samples, urea 120 mg (Supplied by Sigma Aldrich, Gillingham, UK) was also added to the mixture. The mixture was bath-sonicated (30 min) and used immediately after dispersion.

Membrane Electrode Assembly. The catalyst ink was sprayed directly onto the gas diffusion layer (GDL) H23C7 were suppliedFreudenberg (Weinheim, Germany) using an ultrasonic spray system (Sono-Tek ExactaCoat, Sono-Tek Corporation, Milton, NY, USA). The bed of the spray coater was heated to 90 °C during spraying. The flow rate of the Exactacoat was set to 0.4 mL min^−1^, with an offset serpentine spray pattern. A loading of 0.4 mg_Pt_ cm^−2^ was calculated from the mass change on the MEA due to spraying. The GDEs sprayed with urea additives in the ink were soaked face-down in a Petri dish filled with distilled water, after 24 h the GDE was removed from the water and dried. The graphene GDE was assembled together with the Gore Select membrane and a 0.4 mg_Pt_ cm^−2^ HyPlat GDE to act as an anode. The MEAs were hot-pressed at 150 °C and 360 psi. The cell assembly was sealed with a torque of 4 Nm applied to the 12 bolts of the MEA assembly.

### 2.3. Characterisation

The fuel cell testing was performed on a Scribner 850e test system under air/H_2_ with no back pressure and at 100% RH, 80 °C; 1.5/3 was used as the H_2_/O_2_ stoichiometric flow rate. The cell was conditioned by purging with Ar/Ar for 10 min, followed by Ar/H_2_ for 10 min, then air/H_2_ held at OCV for 5 min. The cells were held at 5 A for 1 h, followed by an Ar/H_2_ purge; 25 CV ‘cleaning’ scans were run between 0.06 and 1 V at 20 mV s^−1^. Cyclic voltammetry (CV) measurements were performed between 0.06 and 1 V at 20 mV s^−1^. ‘Fast’ polarisation curves were run at 0.05 V/pt and 10 sec/pt until the current density stabilised. ‘Slow’ polarisation curves were run for data collection purposes at 0.025 V/pt and 30 sec/pt.

TEM was performed on a JEOL 2100 with an accelerating voltage of 100 keV. The samples were briefly bath-sonicated in HPLC-grade ethanol (~10 ug mL^−1^) and drop-cast on lacey carbon copper grids (Agar Scientific, Stanstead, UK).

Raman spectra were recorded with a Renishaw InVia microbeam Raman spectrometer equipped with a 785 nm laser. Measurements were taken directly on the mesoporous carbon, placed on a silicon wafer support, between 1000–2000 cm^−1^, and the data were normalised.

X-ray photoelectron spectroscopy (XPS) was run on a K-Alpha XPS system. The MEA samples were mounted on conductive carbon tape with the top of the GDE pointed upwards and a charge-compensating flood-gun applied to avoid charging effects.

The CL microstructure was analysed by scanning electron microscopy (SEM). Small pieces of the GDEs were embedded in epoxy resin, then the GDE cross-sections were mechanically polished to achieve a mirror-like surface. The observations were performed using a ZEISS MERLIN (Carl Zeiss AG, Oberkochen, Germany) field emission gun scanning electron microscope (FEG-SEM) equipped with two X-ray energy dispersive spectrometry (EDS) detectors. The backscattered electron images and the platinum and fluorine EDS elemental maps were recorded at an accelerating voltage of 4 kV.

The surface areas and pore size distribution were measured through the application of Brunauer, Emmett and Teller (BET) theory, on a Quantachrome Quadrasorb Evo (Anton Paar, St Albans, UK) using nitrogen gas.

## 3. Results and Discussion

The pre-exfoliated FLG nanoplatelets were further exfoliated in order to maximise the exposed surface area by using a previously established reduction methodology [54]. The exfoliation of the FLGs requires that the graphitic sheets be charged through reduction with sodium to facilitate the functionalisation of the FLG with a thioether. These moieties have been reported as having aided in the ‘binding’ of the thiophilic Pt nanoparticles [55,56,57]. The functionalisation of carbon with sulfur-containing organic species has been reported as having improved catalyst durability; however, they have also been reported as having poisoned the Pt catalyst’s surface in some cases [26,58]. The aqueous reduction of chloroplatinic acid was used to deposit ca. 2–5 nm Pt nanoparticles within the indicated diameter range in order to provide the best ORR performance for unalloyed Pt (Figure S1).

Although spraying the CL directly onto the electrolyte membrane has been reported to have improved fuel cell performance, in this work the ink was directly sprayed onto the microporous layer (MPL) of the gas diffusion layer (GDL) [59]. This approach has the advantage of not causing membrane swelling during fabrication and avoiding the warping of the catalyst-coated membrane (CCM) during urea washing steps. The urea content was optimised in preliminary tests, with the best performances obtained at twice the combined weight of the support and catalyst. The lower-urea contents did not have a significant impact on mass transport, while the higher-urea contents resulted in a significantly more heterogeneous CL, loss of mechanical integrity and un-useable MEAs.

The elemental composition of the CLs were estimated via XPS. The standard CL formulation, composed of carbon black/graphene, Nafion and Pt, was found to possess a small nitrogen content, which was likely due to residual DMAc solvent. Urea contains 47 wt% nitrogen, so any significant change in the quantity of urea within the CL was identifiable from the nitrogen content. The nitrogen content in the CL after adding urea to the catalyst ink increased from 0.5 wt% to 2.3 wt%, decreasing to 0.9 wt% after washing (Table 1), indicating the successful removal of a significant portion of the urea. The small apparent nitrogen content remaining after washing was likely due to urea remaining trapped between the graphene sheets in isolated pores. With the quantity of urea added to the CL, the wt% of nitrogen would be expected to have been significantly larger. The fluorine content was also higher than would be expected from a bulk measurement. We suggest that the ionomer that provided the source of the fluorine elements coated the surface of the CL and was preferentially detected by the surface-sensitive XPS measurement. The measured sulfur content was expected due to the functionalisation of the graphene and the sulfonic head group of the ionomer. The fluctuation of Pt and F after the removal of the urea indicated a significant reorganisation of the ionomer network as a result of the soaking in water and subsequent drying. The Raman spectra taken of the sprayed MEAs showed no change in the relative intensity of the D- and G-bands following the inclusion of urea or the washing treatment (Figure S2). This result clearly indicates that the addition of urea to the ink and its subsequent removal did not modify the chemical composition or damage the graphene support.

**Figure 2:**
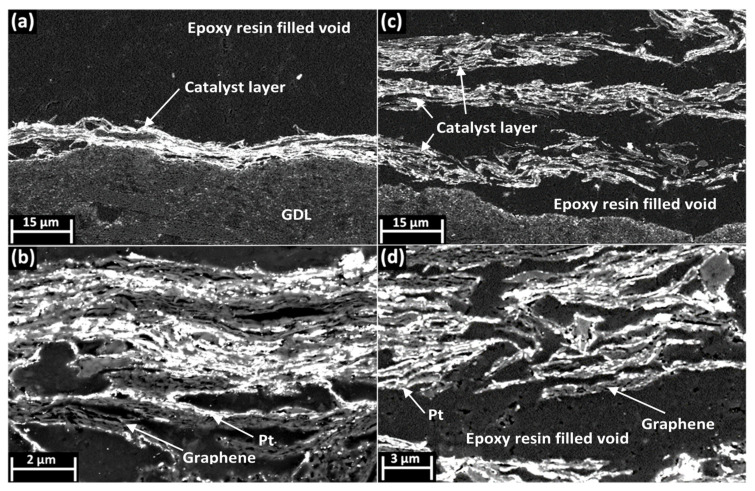
SEM images at two magnifications of graphene-based fuel cell CL cross-sections: (**a**,**b**) SEM images of graphene CLs prepared from a standard ink composition; (**c**,**d**) SEM images of graphene CLs prepared with urea added to the ink before spraying. The different components of the image can be distinguished via the contrast between them (darker, medium grey and brighter contrast are respectively the epoxy resin, graphene or ionomer and the Pt nanoparticles) and are labelled for ease of understanding. The epoxy resin-filled voids were formed as a result of the pore structure within the catalyst layer, and as such were representative of the size and distribution of these pores.

Cross-sectional SEM images of the graphene-based CL without urea (Figure 2a,b) revealed a highly compressed CL morphology with little porosity. Some of the pores appeared to be disconnected from a larger pore network and therefore may have been isolated pores, which significantly limit CL performance (Figure 2a,b) [34]. This apparent lack of mesoscale pores is non-ideal, as it is reported to be essential in order to achieve efficient Pt utilisation and effective mass transport [23,36]. The deposited Pt nanoparticles (observed as chains of bright spots on the outside of the FLG cluster) appeared to be heterogeneously dispersed across the CL, with some regions of the FLG possessing no catalyst. Since the Pt was deposited onto the surface of the FLG, this heterogeneity was most likely due to the incomplete exfoliation of the graphene before the Pt deposition step. The majority of the catalyst nanoparticles in the graphene CL without urea appeared to be trapped between the graphene sheets, with no pore volume between them, reducing Pt utilisation. The CL formed with urea showed a significantly greater pore volume (as shown by the epoxy resin-filled voids) between the FLG clusters; it was therefore facile to observe the uniform Pt coverage on the external surface of these clusters, (Figure 2c,d) which is in good agreement with TEM analysis (Figure S1). Figure 2c,d also shows that after the addition of urea, the CL now contained large macroporous channels (of up to 4 μm) with mesoporous spacing between the FLG layers. The increase in porosity was achieved while maintaining the same Pt loading, resulting in a significantly thicker, although heterogeneous, CL (thickness between 10–50 μm). However, this change in pore structure was accompanied by a significantly greater variation in the film thickness and density across the CL (Figure S6). It is suggested that this resulted from the inhomogeneous crystallisation of urea, which would be expected to create localised regions of higher and lower porosity. Figure 2d shows significantly more Pt catalyst nanoparticles in direct contact with the epoxy resin-filled void rather than other FLG clusters, which would be expected to result in greater Pt utilisation, improved proton and O_2_ transport. The addition of the urea thus significantly changed the pore structure of the catalyst layer, introducing meso- and macropores and improving accessibility to the active Pt NPs.

**Figure 3:**
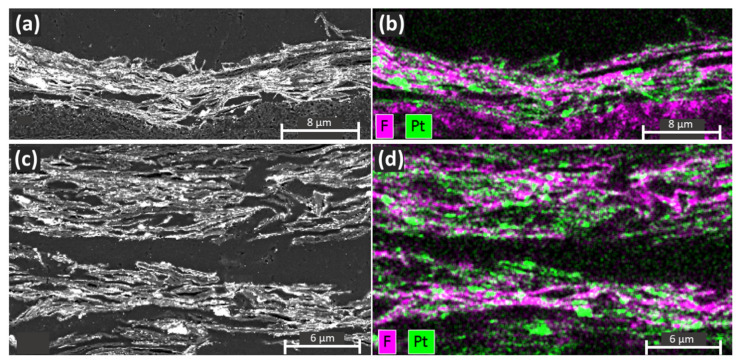
Cross-sectional SEM images and EDS (Pt and F) elemental maps of graphene CLs: (**a**,**b**) SEM image and EDS map of graphene based CL prepared without urea; (**c**,**d**) SEM image and EDS map of graphene-based CL prepared without urea.

Figure 3 shows the distribution of Pt and F across the catalyst layer measured via X-ray energy dispersive spectroscopy (EDS) analysis. The F elemental map shows that for both CLs the ionomer was evenly distributed between the layers of FLG, forming connected pathways across the CL and good contact with the Pt. However, as shown in Figure 3b, the CLs fabricated without urea possessed significant ionomer content, filling the spaces between the layers of restacked graphene. The presence of the Nafion ionomer trapped between the graphene sheets would further be expected to lead to increased O_2_ diffusion limitations [5]. The MEA prepared with urea (Figure 3c,d) possessed more voids between ionomer filled regions while maintaining a highly connected ionomer network, this is expected to reduce overall O_2_ mass transport resistances.

The BET surface area measurements for the graphene-based CLs showed a decrease from 22.2 m^2^ g^−1^ to 11.9 m^2^ g^−1^ due to the addition of urea at the fabrication stage (Figure S7). This value was significantly lower than the theoretical surface area of the graphene, due to restacking and the introduction of the ionomer and catalyst particles onto the surface [60,61]. Given the observed increase in mesoporosity from the SEM (Figure 2), the reduction of the surface area was attributed to a loss in microporosity. The loss of microporosity due to the introduction of the ionomer into the CLs is commonly observed and may represent a more homogenous distribution of ionomer across the entire CL [61]. The PSD measurements of the CLs (Figure S8) seemed to show that the pore volume of the graphene CL was higher at almost every pore width compared to that of the graphene CL with urea. This was in contrast with the higher mesoporosity observed in the SEM images (Figure 2); we therefore suggest that the residual urea content caused an apparent decrease in the calculated surface area and pore volume. It is not easy to quantitively account for this change in pore volume given the unknown content of the urea within the CL and its impact on the pore volume. As such, the pore volumes are reported as normalised (Figure S8), with a greater ratio of larger (meso) to smaller (micro) pores after the addition of the urea.

Measuring the ECSA through the Pt-H peaks in a C-V plot provides an estimate of the electrochemically active surface of Pt nanoparticles deposited onto the electrically conductive carbon and connected to the proton-conducting ionomer network (Figure S9). The ECSA measured for the graphene-based MEAs showed an increase from 11 to 25 m^2^ g_pt_^−1^ following the incorporation of urea into the ink. This is lower than the typical values observed in commercial PtC. This was likely due to the stacked graphene flakes acting as a typical low surface area carbon, leaving the Pt distribution entirely on the exterior of the supports, allowing it to be directly poisoned by the ionomer [23,39,40,41,42]. Since the Pt loading was constant between the carbon black and the graphene samples, the observation of the increased ECSA demonstrated an increased efficiency in Pt utilisation though the improved microstructure. The reduced ECSA for the graphene-based CLs was therefore attributed to the presence of isolated pores.

**Figure 4:**
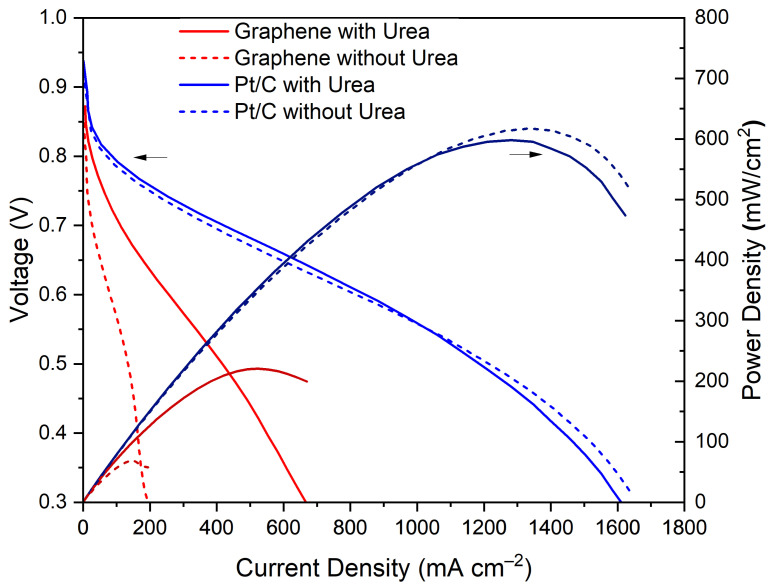
Polarisation and power density curves of graphene-based (in red) and commercial carbon black (in blue), tested in 25 cm^2^ MEAs, under H_2_/air, with no backpressure. The solid lines represent the MEAs that were manufactured with urea, while the dashed lines correspond to the MEAs without urea added to the ink.

Figure 4 shows that the mass-transport-limited current density at 0.3 V of the graphene-based electrodes increased from 190 mA cm^−2^ to 660 mA cm^−2^ after the addition of the urea to the ink. The ECSA increase was also observed at a higher current density in the kinetic region of the polarisation curve. However, the current density change due to the addition of urea was much higher in the low voltage, mass-transport-limited section of the polarisation curve, suggesting that the porosity introduced by the urea additive was highly beneficial to the mass transport of gas across the CL. Since no damage to the FLG support was detected, the significant performance improvement was attributed to the morphological changes observed in the CL (Figure 2). For the commercial Pt/C, there was no significant change in the current density at 0.3 V due to the addition of urea, showing it did not intrinsically poison the Pt catalyst. While the introduction of urea facilitates the creation of pores in the graphene support, and potentially other 2D materials used as catalyst supports, it has little impact on naturally porous bulk supports, such a carbon black.

Typical conditioning did not immediately result in stable performance; the power density increased with each successive polarisation after conditioning, and a significant number (typically between 20–50) of cycles had to be performed before the power density stabilised. This instability was found to be significantly worse if the MEA was not soaked in water before assembly, requiring hundreds of polarisation curves to reach a stable performance. We suggest that the initial inability of the MEAs to reach a stable performance was due to the in situ removal of the excess urea from within the CL in the high-humidity environment. It was found that the initial performance reached equilibrium faster during high-humidity conditioning procedures.

## 4. Conclusions

This article reports a scalable, non-destructive and simply-implemented method for controlling the internal morphology and pore structure of the micro- to mesoscopic length scales of a graphene-based CL. The use of urea additives allowed the one-step fabrication of the CL, followed by a simple and environmentally friendly single-additive removal step. The addition of the urea to the CL ink mitigated the graphene sheets’ (re)stacking during spraying, creating a more porous morphology, increasing the efficiency of Pt utilisation and improving mass transport, leading to significantly higher current densities. The addition of the urea was only effective on graphene-based CLs; traditional Pt/C based on bulk carbon supports did not benefit from the addition of urea. Understanding how the sacrificial additive affects fuel cell performance, including the power density difference between graphene-based CLs and those based on Pt/C, is key to further improving nanomaterial-based electrodes. The technique reported here could be improved by modifying the process for the removal of the additive, as this step often leads to the mechanical breakdown of the CL. Other potentially scalable approaches to urea removal include the use of supercritical CO_2_, or low surface energy solvents; or direct sublimation, in order to allow a greater quantity of the sacrificial additive to be incorporated into the CL. Furthermore, different additives would be expected to have different morphological effects on the CL; moreover, combinations of different additives may allow more precise control over the PSD and porosity of CLs.

## Figures and Tables

**Table 1:** Elemental composition of the graphene-based MEAs calculated from XPS results. MEAs were sprayed with urea added to the ink and no urea for comparison; in addition, XPS data were obtained for the urea-sprayed MEAs before washing. XPS spectra are provided in Supplementary Materials (Figures S3–S5).

Weight%	C 1s	N 1s	O 1s	F 1s	S 2p	Pt 3d
No Urea	24.9	0.5	6.6	60.8	2.6	4.5
Urea	25.2	2.1	6.4	48.4	1.4	16.6
Urea and washed	27.2	0.9	6.4	55.9	2.8	6.4

## Data Availability

The data presented in this study are available in the article or supplementary material.

## Supplementary Materials

The following are available online at https://www.mdpi.com/article/10.3390/nano11102530/s1, Figure S1: Transmission electron microscopy images of platinum catalyst deposited onto graphene showing particles between 3 and 5 nm, Figure S2: Raman spectra of graphene based catalyst layers when deposited without urea, with urea and with urea after washing, Figure S3: Survey XPS of graphene based catalyst layer fabricated without urea, Figure S4: Survey XPS of graphene based catalyst layer fabricated with urea before washing, Figure S5: Survey XPS of graphene based catalyst layer fabricated with urea, Figure S6: Cross sectional SEM images of several sections of the CL after the removal of urea showing it’s inhomogeneous nature, Figure S7: The pore surface area distribution of graphene based CLs and those fabricated with urea, Figure S8: The pore size distribution of graphene based CLs and those fabricated with urea, Figure S9: Cyclic voltammetry of graphene and commercial carbon black MEAs fabricated with and without urea.
