# Supplementary material for: Scalable Sacrificial Templating to Increase Porosity and Platinum Utilisation in Graphene-Based Polymer Electrolyte Fuel Cell Electrodes
Source: Nanomaterials (Basel). 2021 Sep 28;11(10):2530. doi: 10.3390/nano11102530 (PMC8539662; doi:10.3390/nano11102530)
Supplement: Supplementary file 1 [file nanomaterials-11-02530-s001.zip › nanomaterials-1304910-supplementary.pdf]

Supplementary Materials

Scalable Sacrificial Templating to Increase Porosity and Platinum Utilisation in Graphene-based Polymer Electrolyte Fuel Cell Electrodes

Theo A. M. Suter [1,*], Adam J. Clancy [2,3], Noelia Rubio Carrero [4,5], Marie Heitzmann [6], Laure Guetaz [7], Paul R. Shearing [1], Cecilia Mattevi [5], Gérard Gebel [7], Christopher A. Howard [3], Milo S. P. Shaffer [4,5], Paul F. McMillan [2] and Dan J. L. Brett [1,*]

1. Electrochemical Innovation Lab, Department of Chemical Engineering, University College London,
London WC1H 0AJ, UK; p.shearing@ucl.ac.uk
2. Department of Chemistry, University College London, London WC1H 0AJ, UK; a.clancy@ucl.ac.uk (A.J.C.); p.f.mcmillan@ucl.ac.uk (P.F.M.)
3. Department of Physics and Astronomy, University College London, London WC1H 0AJ, UK;
c.howard@ucl.ac.uk
4. Department of Chemistry, Imperial College London, London SW7 2AZ, UK; n.rubio-carrero@imperial.ac.uk (N.R.C.); m.shaffer@imperial.ac.uk (M.S.P.S.)
5. Department of Materials, Imperial College London, London SW7 2AZ, UK; c.mattevi@imperial.ac.uk
6. Université Grenoble Alpes, CEA, Liten, DEHT, 38000 Grenoble, France; marie.heitzmann@cea.fr (M.H.)
7. Université Grenoble Alpes, CEA, Liten, DTNM, 38054 Grenoble, France; laure.guetaz@cea.fr (L.G.); gerard.gebel@cea.fr (G.G.)

* Correspondence: theo.suter.14@ucl.ac.uk (T.A.M.S.); d.brett@ucl.ac.uk (D.J.L.B.)

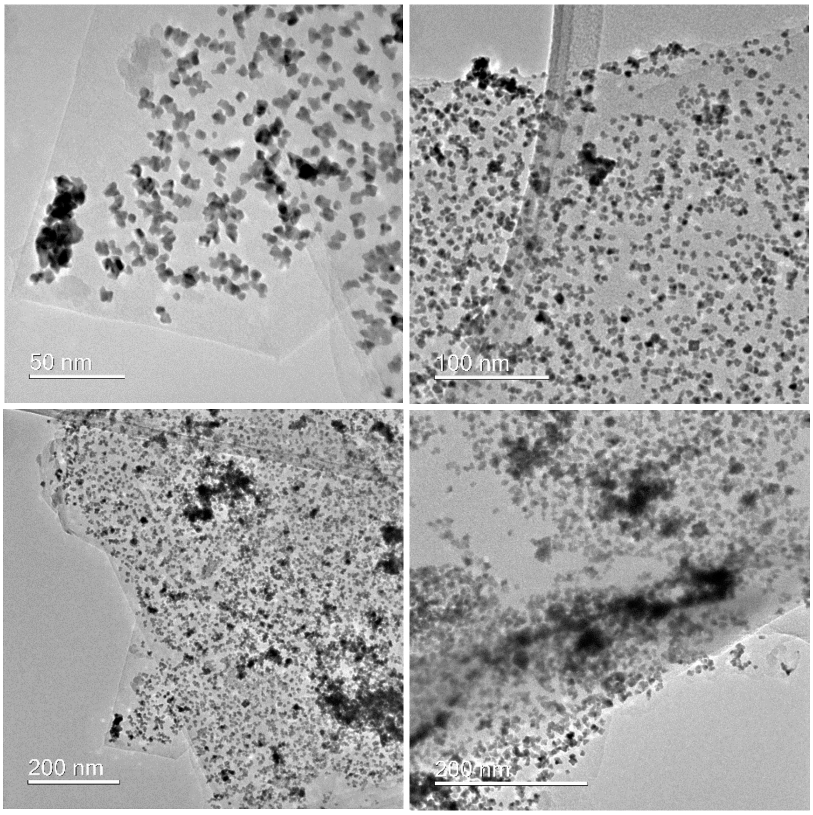

Figure S1. Transmission electron microscopy images of platinum catalyst deposited onto graphene showing particles between 3 and 5 nm.

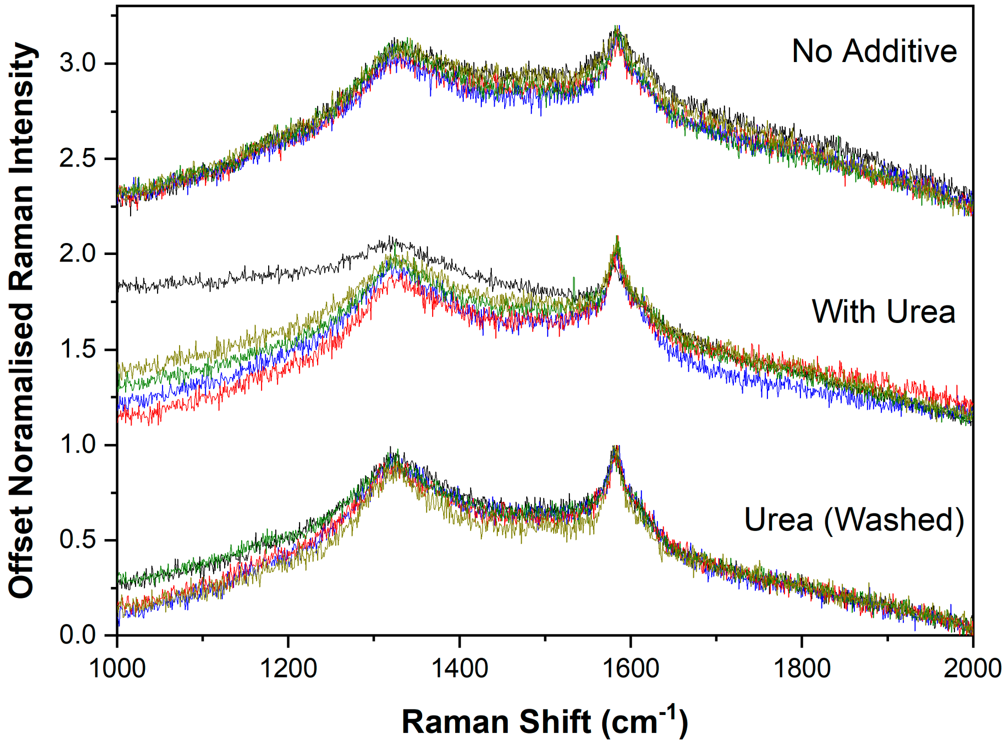

Figure S2. Raman spectra of graphene based catalyst layers when deposited without urea, with urea and with urea after washing.

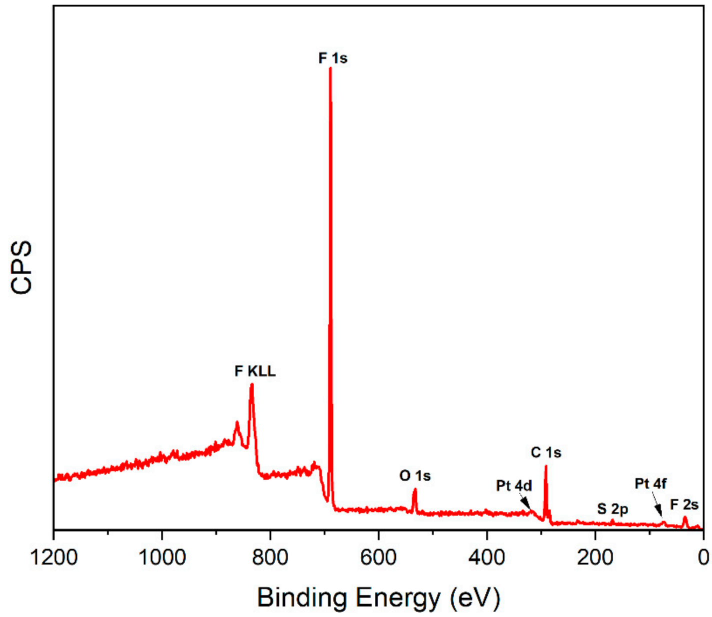

Figure S3. Survey XPS of graphene based catalyst layer fabricated without urea

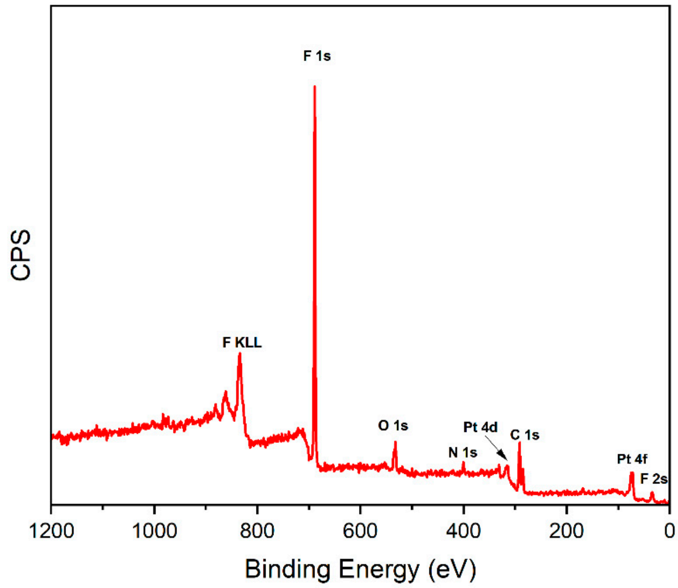

Figure S4. Survey XPS of graphene based catalyst layer fabricated with urea before washing

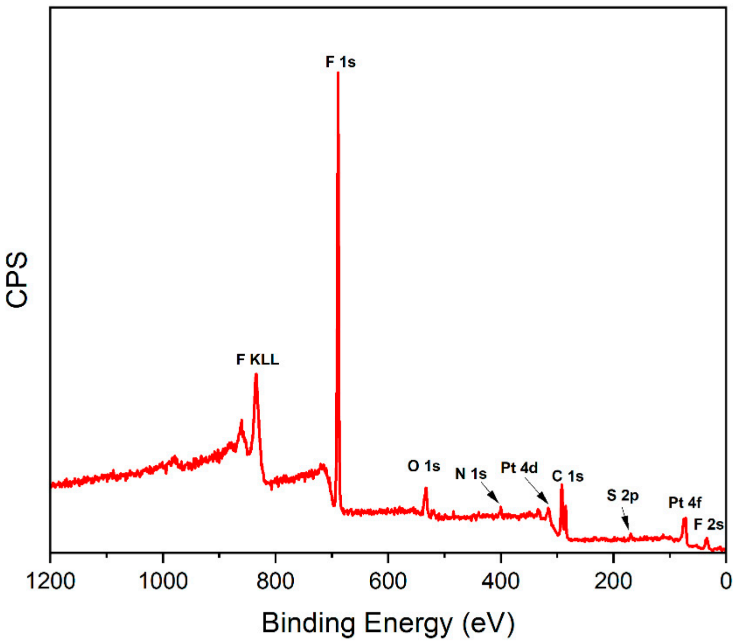

Figure S5. Survey XPS of graphene based catalyst layer fabricated with urea

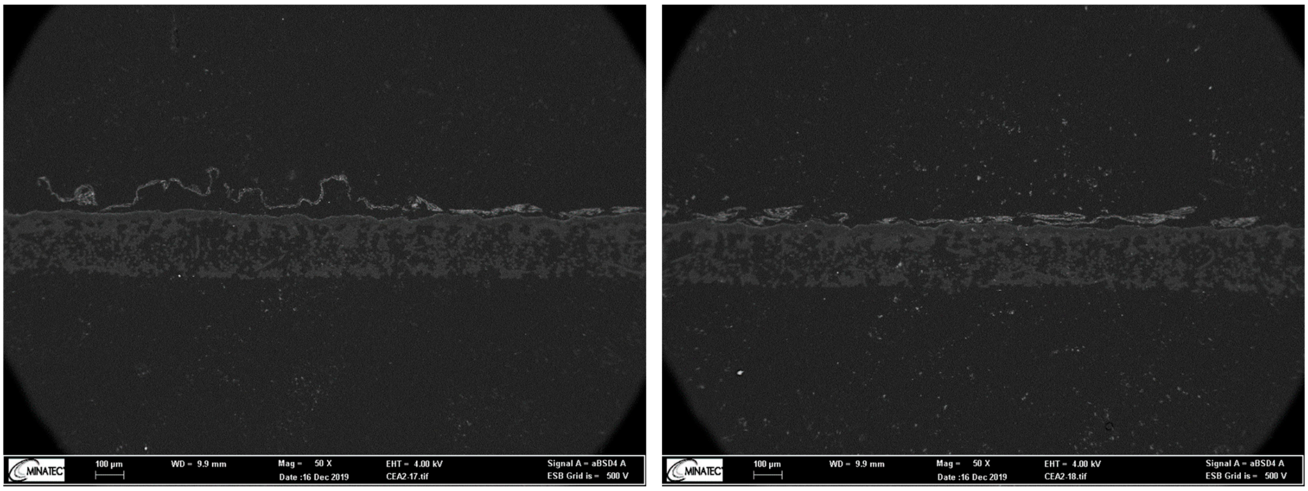

Figure S6. Cross sectional SEM images of several sections of the CL after the removal of urea showing it's inhomogeneous nature.

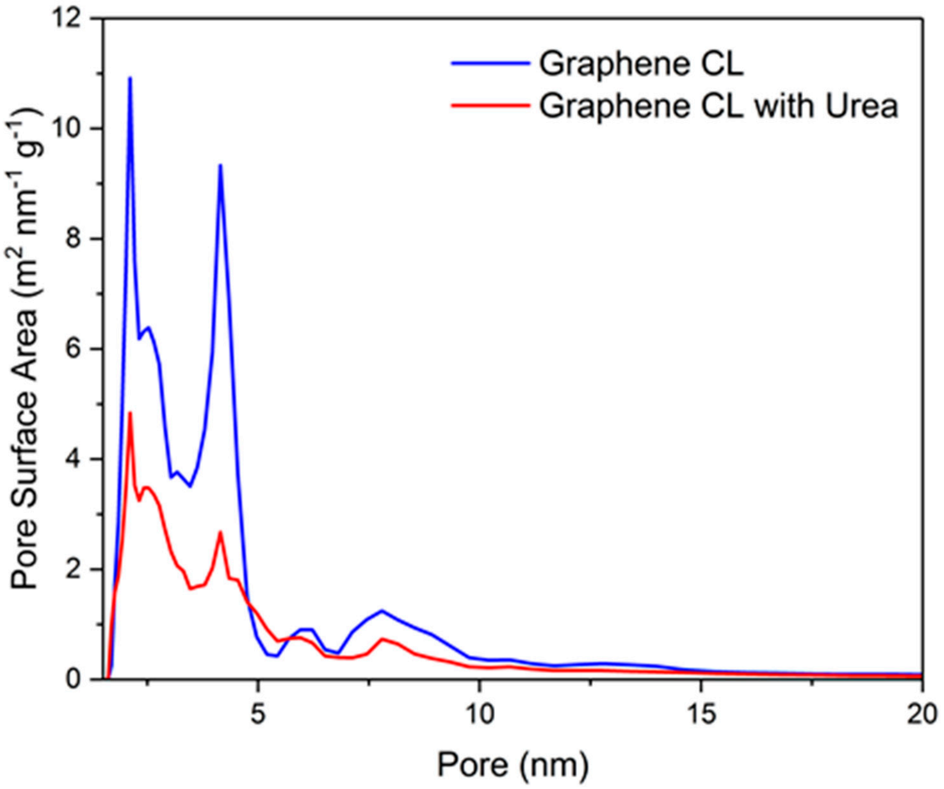

Figure S7. The pore surface area distribution of graphene based CLs and those fabricated with urea.

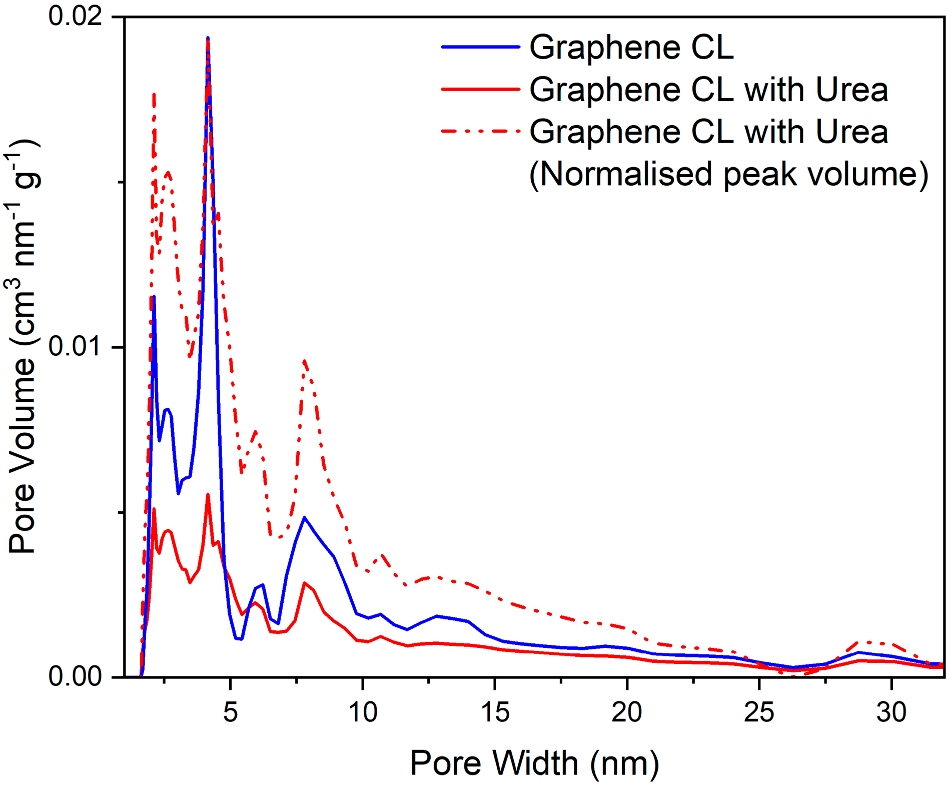

Figure S8. The pore size distribution of graphene based CLs and those fabricated with urea. The relative change in pore volume at different pore width is demonstrated by normalised graphene CL with urea curve.

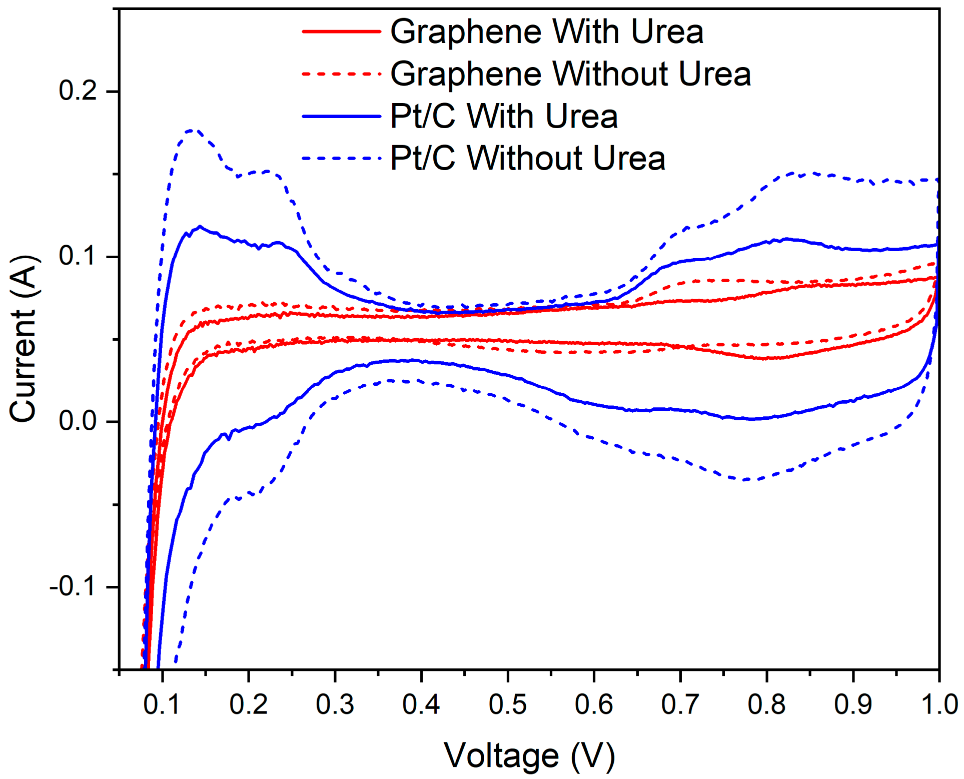

Figure S9. Cyclic voltammetry of graphene and commercial carbon black MEAs fabricated with and without urea.
